# Improving continuity by bringing the cancer patient, general practitioner and oncologist together in a shared video-based consultation – protocol for a randomised controlled trial

**DOI:** 10.1186/s12875-019-0978-8

**Published:** 2019-06-25

**Authors:** Theis Bitz Trabjerg, Lars Henrik Jensen, Jens Søndergaard, Jeffrey James Sisler, Dorte Gilså Hansen

**Affiliations:** 1 National Research Center of Cancer Rehabilitation, Research Unit of General Practice, University of Southern Denmark, J.B. Winsloews Vej 9A, 5000 Odense C, Denmark; 2 Department of Oncology, Lillebaelt Hospital, Vejle, Denmark; 3 Department of Family Medicine, Faculty of Health Sciences, University of Manitoba, Manitoba, Canada; 4 Danish Colorectal Cancer Center South, Center of Clinical Excellence, Vejle Hospital, Institute of Regional Health Research, University of Southern Denmark, Odense, Denmark

**Keywords:** Cancer, General practice, Video-consultation, Randomised controlled trial, Patient-centeredness

## Abstract

**Background:**

Strengthening the coordination, continuity and intersectoral cooperation for cancer patients’ during cancer treatment is being underlined by international guidelines and research. General practitioners have assumed a growing role in the cancer patient disease trajectory because of their roles as coordinators and the consistent health provider. However, general practitioners are challenged in providing support for cancer patients both during treatment and in the survivorship phase. General practitioners reported barriers are lack of timely and relevant communication from the oncologist and limited knowledge to guidelines, as well as lack of trust from patients.

Therefore, the current study will examine whether a shared video-based consultation between the cancer patient, general practitioner and oncologist can ease general’ challenges and thereby enhance the patient-centeredness for the cancer patients and their perception of intersectoral cooperation and continuity.

**Methods:**

The study is designed as a pragmatic randomised controlled trial for patients starting chemotherapy at the Department of Oncology, Lillebaelt Hospital, Denmark who are listed with a general practitioner in the Region of Southern Denmark. We intend to include 278 adults diagnosed with colorectal, breast, lung, gynecologic or prostate cancer.

The intervention group will receive the “Partnership intervention” which consists of one or more video-consultations between the cancer patient, general practitioner and oncologist. The consultations are estimated to last between 10 and 20 min. The specific aims of the consultation are, summary of the patient trajectory, sharing of knowledge regarding comorbidity, psychosocial resources and needs, physical well-being, medicine, anxiety and depression symptoms, spouses, workability and late complication and side-effects to the cancer treatment.

**Discussion:**

Video-based consultation that brings the cancer patient, the general practitioner and the oncologist together in the early phase of treatment may facilitate a sense of partnership that is powerful enough to improve the patient’s perception of intersectoral cooperation, continuity of cancer care and health-related quality of life.

**Trial registration:**

ClincialTrials.gov Identifier: NCT02716168. Date of registration: 03.03.2016.

**Electronic supplementary material:**

The online version of this article (10.1186/s12875-019-0978-8) contains supplementary material, which is available to authorized users.

## Background

With the current rates of new cancer cases and the increase in survival among cancer patients [1], there is a need to improve the lives of cancer patients. International guidelines have underlined the importance of strengthening the coordination and continuity of care during and following cancer treatment. Each cancer patient should be involved in decision-making [2, 3] and informed about which healthcare providers are taking care of their individual needs [4].

When receiving a cancer diagnosis, patients are often faced with psychological distress and loss of control [5]. They can be overwhelmed by the diagnostic process, difficult treatment decisions, ensuring treatment and side effects [5]. Understanding the detailed information is challenging [6], and patients’ might have difficulties even identifying which care provider is supposed to help with a specific problem [4, 7]. Studies have shown that patients who feel poorly informed may feel more vulnerable [8]. Therefore, new organisational strategies are needed.

Interventions focusing on gaps in continuity of cancer care are expected to have an impact on the quality of care [9]. Furthermore, improved continuity of care may be associated with lower health care needs [9]. Several studies have underlined gaps in continuity of care like a Canadian survey of patients who have colorectal cancer. More than 50% of the patients did not feel they had been fully informed about resources, support and educational materials to help consolidate continuity of care [4].

General practitioners (GPs) are assuming a growing role as coordinators of the cancer patients’ disease trajectory to enhance continuity [10]. However, GPs are challenged in providing support for cancer patients both during treatment and in the survivorship phase. Reported barriers include a lack of timely and specific communication from the oncologist, lack of trust from patients and limited knowledge to guidelines [11].

Within the Danish healthcare setting, trials have aimed to improve shared decision-making and continuity of cancer care across healthcare sectors during cancer treatment [12–14]. Despite rigorous designs and adequate statistical power, these studies did not identify statistically significant differences in the intervention groups.

Face-to-face communication is important for patient satisfaction and their comprehension of the information received. Furthermore, studies have shown that seeing each other is essential for the relationship [15–17]. Therefore, we hypothesised that bringing the GP and oncologist face-to-face in a shared video-consultation with the cancer patient might address some of the problematic issues for the patient, and enhance their perception of continuity of care. Due to geographical reasons and time constraints, a joint consultation between patient, GP and oncologist is not feasible as part of routine cancer care. Video-based communication may be an alternative solution [17–22]. Studies from the cancer setting have shown that patients can experience effective communication and relationship with their doctor through technology-based consultation [17].

The study is designed as a pragmatic randomised controlled trial (RCT) [23, 24] allocated to an intervention and a control group in a 1:1 ratio. The GPs and oncologists may have patients in both groups.

We hypothesise that bringing the oncologist and GP together with the patient in a single shared video-based consultation in the early months of treatment, in comparison with usual care, will 1) increase the cancer patients’ perception of intersectoral cooperation (primary outcome) and 2) increase their perceptions of continuity of cancer care, distress and health-related quality of life (secondary outcomes).

## Methods

This study is designed and reported according to the Medical Research Council guidance on complex interventions [25, 26] and the SPIRIT-PRO guideline [27]. The SPIRIT-PRO-Checklist can be found under additional file 1.

### Methods: participants, interventions and outcomes

#### Study setting

The study will be performed in cooperation between the Department of Oncology, Lillebaelt Hospital, Denmark and general practices in the Region of Southern Denmark. Annually, around 1.300 patients are referred for chemotherapy at this department. The department endeavours to ensure that patients see the same “most responsible physician” at each visit to ensure greater continuity and a better outcome for patients [24].

The hospital’s catchment area includes about 300.000–750.000 citizens depending on the cancer diagnosis and 500 general practitioners working in approximately 300 general practices medical centres comprised of 1–8 physicians. In Denmark, all citizens are eligible for free medical service at public hospitals and in the primary care sector. The GPs are gate-keepers to the more specialised health care services, and more than 98% of the population is enrolled in a specific general practice clinic [28].

#### Eligibility criteria

All newly diagnosed cancer patients 18 years of age and older are eligible if receiving treatment with chemotherapy for the first time at the Department of Oncology, Lillebaelt Hospital, Denmark for any cancer diagnosis. Participants must have an expected survival time of more than 7 months as assessed by an oncologist, and be able to speak and read Danish.

#### Technical issues

Video-based consultations require easy-to-use, high quality, reliable, safe and legal communication equipment. We chose the Cisco®, TANDBERG™ E20 screen system already in use at the hospital for interpreting services and by roughly 30% of the GPs in the study area. Participating GPs with patients in the intervention group will be offered a free set up and support by the Health innovation Centre of Southern Denmark.

#### Intervention

##### Control group

The control group receives ‘usual care’ regarding the exchange of information between the Department of Oncology and primary care. ‘Usual care’ includes an electronic summary letter to the GP after each visit to the Department of Oncology. In case of questions, the GPs can always telephone the hospital and vice versa. Furthermore, patients’ can freely contact their GP or a specific coordinator at the Department of Oncology.

##### Intervention group

In addition to ‘usual care’, patients in the intervention group will receive ‘the partnership intervention,’ which brings the cancer patient, general practitioner and oncologist together in a shared video-consultation. The consultation is expected to last 10 to 20 min and is chaired by the oncologist. The consultation is planned as early as possible and within 12 weeks from the time of inclusion. More consultations can be planned within 6 months if the GP, oncologist or patient request.

Patients’ are allowed to sit in the office of either the oncologist or the GP. The oncologist and the GP receive specific information about the aim of the consultation including a list of relevant subjects based on the literature and pilot testing (see Table 1). The oncologist makes a summary of the consultation into the hospital electronic patient record and sends it to the GP. The summary is available for the patient at Sundhed.dk, an online portal where patients in Denmark can read their entire medical record from secondary care.

**Table 1:** Possible topics for the video-consultation

• A Short summary of the patient trajectory	
• Sharing of knowledge regarding comorbidity	
• Psychosocial resources and needs	
• Agreements on who should take care of what and when in the future	
• Physical well-being	
• Medicine	
• Anxiety and depression symptoms	
• Spouses	
• Workability	
• Late complication and side effects to the treatment	
• Other	

#### Outcomes and instruments/scales

##### Primary outcome with a description of the scale

The primary study outcome is the single item “global assessment of inter-sectorial cooperation,” which is part of the Danish questionnaire “*Patients’ attitude to the health care service”* [13] measured 7 months after the enrolment of the patient (additional file 2). This 26-item questionnaire was based on the English questionnaire *“The patient career diary”* [29]. The Danish adaptation was based on interviews with Danish cancer patients and caregivers [30] and the questionnaire template of the English version [29].

Besides our primary outcome, this questionnaire also includes two other single items and five index scales, seeking to address the patient’s attitude towards the cooperation in the health care system. All items have a recall period of 3 months and are answered on a five-point Likert scale from strongly agree (1) to strongly disagree (5). According to the scoring manual, all index scales and items are linearly transformed to values 0 to 100 with higher numbers meaning higher disagreement levels [31].

##### Secondary outcomes - patients

Secondary outcomes address patient perceptions of health-related quality of life, cancer care coordination, illness intrusiveness, satisfaction with information, and depressive and anxiety symptoms.

1) Health-related quality of life assessed by the 30-item European Organization for Research and Treatment Quality of Life Questionnaire C-30 version 3.0 (EORTC QLQ C-30, 32]. It has a recall period of 2 weeks, for patients with various types of cancer [32]. Each item is scored from one to four according to ‘not at all’ (1) to ‘very much’ (4), except for the global health status that is scored very poor (1) to excellent (7). Mean scores are linearly transformed to 0–100 scores. High scores represent healthy functioning in the functioning scales and global health status, but a high level of problems for symptom scales and single symptom items.

2) The Australian Cancer Care Coordination Questionnaire (CCCQ) assesses the patient perception of the coordination of cancer care with a recall period of 3 months [33]. Based on 22 items, the CCCQ includes a total score (item 1–20, ranging 20–100), two single items (ranging 1–10) and two subscales covering communication (item 1–13) and navigation (item 14–20) ranging 13–65 and 7–35, respectively. Answers are given on five-point Likert scales from never (1) to always (5) except the global items using a ten-point Likert scales from very poor (1) to very good (10).

3) The Illness Intrusiveness Rating Scale (IIRS) has 13 items assessing the extent to which illness or its treatment interfere with different aspects of life [4, 34]. The questionnaire does not have a defined recall period. Answers are given on a seven-point Likert scale ranging from 1 (not very much) to 7 (very much). For data analyses, a total score ranging from 13 to 91 is generated by addition of all 13 items. In addition to the total score, three subscales scores can be calculated. They range from one to seven represented by subscale means. A higher score indicates increased illness intrusiveness.

4) EORTC Information Questionnaire 25 (EORTC INFO 25) [35] has 25 items that cover several aspects of patient satisfaction with the information provided during cancer treatment. No specific recall period is described. It includes a total score, four sub-scales and four single dichotomous items (yes or no). Beyond, the single items answers are given on a four-point Likert scale from not at all (1) to very much (4). According to the manual, all scores are linearly transformed to a 0–100 scale [35].

5) The Patient Health Questionnaire (PHQ-9) is designed for screening of depression in non-psychiatric settings based on nine items answered on a four-point Likert scale [36]. The recall period is 2 weeks, and the sum score ranges from 0 (no depressive symptoms) to 27 (all symptoms occur daily). Values above ten should alert physicians of a significant depression requiring active intervention (sensitivity of 80% and a specificity of 92% for DSM-IV major depression [37]). Additionally, we use The Generalised Anxiety Disorder seven scale (GAD-7, [38]) to assess anxiety symptoms based on seven items and the same two weeks recall period and same four-point Likert scale as described for the PHQ-9. The GAD-7 can be used as a screening tool with a sum score ranging from 0 to 21. A score of ten or higher should alert physicians’ of a generalised anxiety disorder.

##### Secondary outcomes - GPs

All GPs in the control group and GPs in the intervention group will receive a questionnaire 4 months after the enrolment of the patient. They are asked to assess their satisfaction with own contribution to patient health care including treatment of comorbidities, the relevance of patient visits in general practice, the experience of overall coordination of treatment, as well as communication and cooperation with the oncological department. Given the lack of standardised scales to assess GP perceptions, we devised ad hoc questions.

##### Other variables

Information on healthcare-seeking in general practice and the department of oncology (contact form, date and purpose of visit, number of admission to hospital and number of days hospitalised) will be retrieved from electronic patient files for an economic evaluation.

Beside, the baseline questionnaire completed by patients will include demographic and clinical information about age, cancer type, cancer recurrence status, previous surgery, treatment, occupation, workability, education and family relationship including children and if they are living at home.

##### Translation of questionnaires

For this study, the CCCQ and the IIRS were translated from English to Danish based on the guidelines for the process of cross-cultural adaptation of self-report measures proposed by Beaton et al. [39]. The translation procedures were summarised in separate reports using the report template proposed by Beaton et al. [39].

#### Participant timeline for patient-reported outcomes

Participants will receive a questionnaire at baseline and four and seven months after inclusion (Table 2). Demographic information will be asked only at baseline. Otherwise, the questionnaires will contain the same scales and items presented in the same way. Generally, chemotherapy lasts six-seven months. The follow-up times at four and seven months should allow assessment, twice after the intervention, one in the middle of the treatment and one at or near the end of treatment, in most cases.

**Table 2:** Enrolment, interventions, and assessments schedule

		STUDY PERIOD						
	Enrolment	Allocation			Post-allocation			
TIMEPOINT	*-t*_*1*_	0	*t*_*1*_	*t*_*2*_ *	*t*_*3*_ *= 3–12 weeks*	*t*_*4*_ *= 4 months*	*t*_*5*_ *= 4–6 months*	*t*_*6*_ *= 7 months*
ENROLMENT:								
Eligibility screen	X							
Informed consent	X							
*Contact to general practice intervention group*	X		X					
*If necessary IT install at general practice*				X				
Allocation		X						
INTERVENTIONS:								
*Partnership Video Consultation Intervention*					X		(X)**	
*Control Group*								
*GP Video Consultation Intervention*					X			
ASSESSMENTS:								
*Baseline variables patients*	X							
*Patient intervention group*					X***	X	(X)***	X
*Patient control group*						X		X
*GP intervention group*					X****	X	(X)****	
*GP control Group*						X		
*Oncologist*					X*****		(X)*****	

*Shortly after inclusion of the patient the GP in the intervention group is contacted to give their consent. ** The possibility for additionally video consultation if one of the involved parts request it. ***Patient in the intervention group evaluate the video consultation using PROMs. **** GPs in the intervention group evaluate the video consultation using ad hoc questions. ***** Oncologist in the intervention group evaluate the video consultation using ad hoc question

In addition to the between-group analysis, patients, GPs and oncologists in the intervention group will be asked to complete a short questionnaire after the video-based consultation, covering their experiences of the consultation. These will not be described in further detail in this paper.

#### Sample size

We based our sample size assumptions on knowledge from a Danish intervention study of cancer patients where our primary outcome was used [13]. This study estimated mean values of 56.6 and 69.6 for the control and the intervention group, respectively, and a common standard deviation of 27. Based on these estimates and a clinically relevant difference of 20% between the groups, we calculated a sample size of 194 (2 × 97) participants, with a 0.05 two-sided significance level and a 10% risk of type II error, i.e. a power of 90%. However, as we expect dropout of 30%, our target is to recruit 278 patients.

#### Recruitment strategies for achieving adequate patient enrolment

Timely recruitment of patients will require strong support and managerial endorsement in the hospital department. Furthermore, diligent oversight by project personnel will be crucial to keep track of the many logistical steps, stakeholders, general practices, cancer teams, patients and technical solutions at play in this study. To achieve patient enrolment as planned, one project nurse will head a small team of research nurses responsible for the practical issues around the enrolment of patients.

#### Feasibility

The study was tested in a pilot study before study start to help implement this pragmatic randomised controlled trial in routine clinical practices. The pilot study examined previously identified key uncertainties to support the refinement of the study design [26]. The primary foci of our pilot study performed in January to April 2016 were: 1) assessment of the willingness of patients and GPs to participate 2) patient and GP perspectives on the oral and written study invitation (readability, usefulness and emotional strain), 3) time required by patients to complete various aspects of the study including invitation, baseline questionnaire, and the video-based consultation, 4) administrative, logistical and technical procedures for inclusion of patients, invitation of GPs, and scheduling and delivery of the common video-based consultation, 5) users’ perspectives on acceptability of a video-based consultation, including patients’, GPs’ and oncologists’ perspectives, and 6) usefulness of the consultation guideline. We learned a lot from the pilot phase and changed the study procedures and design in accordance [26].

Fidelity of the intervention is another crucial step in ensuring the validity of our outcomes. Engagement and training of GPs and oncologists for patients allocated to the intervention group are therefore crucial. Since we anticipate that it may be challenging to contact GPs, we will assign one project nurse to these tasks. She will be engaged at the Research Unit of General Practice, Odense and will work in close collaboration with the project manager (TBT) and the team of nurses at the department.

### Methods: assignment of intervention

#### Allocation (Fig. 1)

Participants will be recruited at the Department of Oncology, Lillebaelt Hospital. Firstly, eligibility will be verified in the electronic patient record. Clinic nurses will then ensure that patients are informed and invited to participate at their next appointment.

**Fig. 1:**
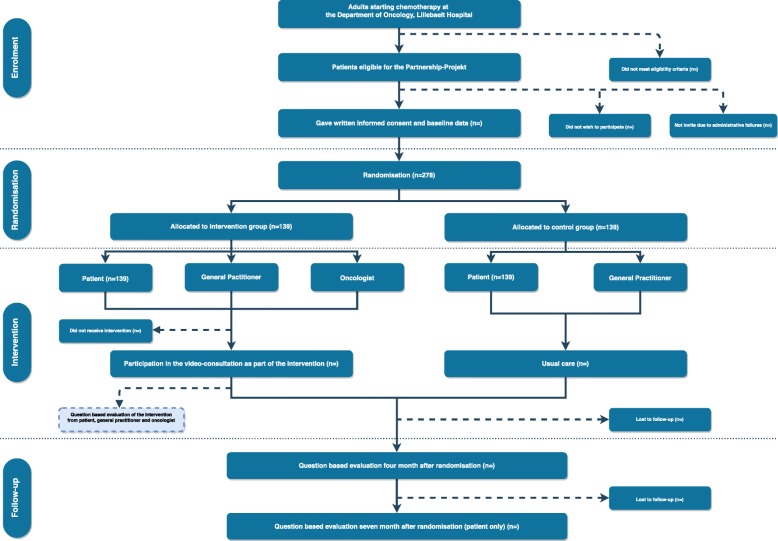
Study flow

After completion of the written informed consent and baseline questionnaire, the clinical nurses responsible for obtaining consent contacts the project nurse responsible for allocation by telephone. She will identify the allocation code in RedCap® [40] and inform the patient.

We will use block-randomisation with different block sizes and sequences to assure the best possible balance between groups. Only the RedCap® [40] data manager from the collaboration partner Odense Patient Data Explorative Network, Department of Clinical Research, University of Southern Denmark and Odense University Hospital (OPEN) will know the block sizes and sequences. No strong evidence regarding relevant stratification factors for the primary outcome is available. Thus, the randomisation will not be stratified. However, the influence of age, gender, education and cancer type will be investigated in exploratory analyses.

GPs will be invited when one of their patients is enrolled in the project. GPs are themselves not allocated and may, therefore, be enrolled several times and to both groups. In case the patient is in the intervention group, the GP is contacted immediately after enrolment, whereas the GP in the control group is first contacted after four months.

#### Blinding

For obvious reasons, patients, GPs nor oncologists in the intervention group are blinded to the patient’s allocation status. Unless told by the patient, GPs in the control group are blinded until they receive the four-month questionnaire. Until the end of the analyses, researchers will be blinded to allocation, except for the project manager (TBT).

### Methods: data collection, management and analysis

#### Plan for assessment and collection of outcomes (Table 2)

Patients will fill in a paper-based baseline questionnaire before randomisation, and electronic follow-up questionnaires will be sent to them at the email address obtained at enrolment. For GPs in the intervention group, questionnaires are collected electronically, while GPs in the control group are sent paper-based questionnaires with a return, stamped envelope.

All electronic questionnaires will be managed using the software program RedCap® [40], which is capable of automatically emailing secured links with questionnaires and retrieving answers online. In all cases, patients and GPs can ask for a paper-based version including a prepaid return envelope.

#### Patient burden

Based on piloting we know that the questionnaire is acceptable to patients concerning readability, time to complete, and emotional strain. Completion for patients may take up to 1 hour.

#### Retention to follow-up

For each iteration of the surveys, RedCap® [40] will automatically email reminders for the follow-up questionnaires twice with 2 weeks intervals. By postal service, a third and last reminder is sent with a prepaid return envelope.

#### Language

All study material and questionnaires are in Danish. The capability of reading Danish is an eligibility criterion, and all working physicians can speak and read Danish.

#### Plan for data if a participant discontinues

All data will be analysed following the intention-to-treat principles [41], including cases where the GP declines to participate, and no intervention takes place. In case of patient withdrawal of consent, death or relocation, cases will be withdrawal.

#### Data management

##### Data entry

Data from the electronic questionnaires are entered RedCap® [40] automatically while paper-based data are entered twice by a secretary at the Research Unit of General Practice using double data entry.

##### Data security and storage

By Danish law baseline questionnaires in paper form are stored in secured lockers at the Research Unit of General Practice, and all data retrieved by RedCap® [40] are stored at a secured server housed by OPEN.

#### Statistical methods

For the between-group comparison of the primary outcome, we will use a standard T-test for the mean difference between two independent means. As a supplementary analysis, we will apply the Mann-Whitney test instead.

Secondary outcomes will be handled similarly, and always according to available manuals. Dichotomous data will be analysed using Fisher’s exact tests and to correct for the multiple comparisons we will apply the Bonferroni correction.

Missing data will be analysed using two strategies - complete-case analysis with only patients with non-missing scores and an imputation approach following the respective manuals. Before imputation, we will investigate if the data are missing randomly or not. Missing data of the EORTC QLQ C-30 and EORTC QLQ INFO-25 will be handled as described in the manual [42].

### Methods: monitoring

#### Monitoring

During the trial, there will be no monitoring of data by researchers or its sponsors, except when sending out reminders. No interim analysis is planned.

#### Harms and description of auditing if any

No harm to patients or other study participants is expected. Unexpected harmful, uncomfortable or abrasive experiences can be communicated verbally or in writing to the project group as stated in the information material given at study inclusion.

### Methods: patient and public involvement

The project idea was conceived in a working group called “Doctors’ Partnership” at the Lillebaelt Hospital, Vejle, Denmark. Representatives from the Patient and Relatives Council at the Hospital participated in the working group. Furthermore, the council have proofread the information material and questionnaires for the study. In the pilot study, the burden of the intervention and questionnaires was assessed by patients and were found acceptable. There are no plans to involve patients in recruitment and conduct of the study, besides in the development phase and interpretation of the results.

We are planning to host a workshop with the Patient and Relatives Council to discuss the results from the study and its implications for cancer patients. Final results will be available for patients and the public at the project website, as stated in the information material.

### Ethics and dissemination

#### Permissions and registrations

The Regional Ethics Committee on Biomedical Research in Denmark (S-20142000-138) and the Danish Data Protection Agency (2014-41-3534) have approved the study. Collection of data will be handled according to national law restrictions. The study is indexed at https://www.clinicaltrials.gov (NCT02716168), registered 03 March 2016.

#### Plan for communication important protocol modifications

Modification to the protocol will be announced in Danish at the project website http://www.sdu.dk/psp and in English at https://www.clinicaltrials.gov.

#### Who will obtain informed consent?

The outpatient clinic nurses at the Oncological Department will obtain informed consent from patients. The consents forms are stored at the Department of Oncology, clinical research unit.

The project nurse responsible for GP participation will obtain oral consent from GPs in the intervention group.

GPs in the control group will be informed about the study at the four-month questionnaire but are not requested to return a consent form.

#### Confidentiality

Assessment and information about potential participants are kept in the hospitals electronic patient record. At the eligibility assessment before inclusion, only the physician responsible for the treatment and the nurse manager at the department has access to the electronic patient record. After informed consent from the patient, the project manager (TBT) has access to the electronic patient record.

#### Access to data

Data access is restricted to the research group including the statistician.

The project nurses do not have access to obtain data from the project database in RedCap® [40]. They can only view data in connection with enrolment and view data necessary for project management. All spreadsheets in RedCap® [40] are logged. At the end of the analytical phase of the study, all electronic data will be transferred to the Danish National Archives.

#### Provisions

GPs in the interventions group will receive a fee for the video consultations, which will be 48 or 97 Euros for up to 30 min and above 30 min, respectively. GPs will receive 18 euros for filling in a questionnaire at 4 months.

Patients and oncologist will not receive any reimbursement.

#### Dissemination policy

Trial results will be published in peer-reviewed journals, discussed at national and international conferences and communicated for the public at the project website http://www.sdu.dk/psp.

#### Ethical considerations about the trial

Data security when conducting video-consultation is essential. Patients show a high degree of trust in regards to data security because they trust the healthcare staff using the technology. Therefore, a high level of data security is needed, and all video-consultations are held on the Region of Southern Denmark secure videoconference servers with the use of virtual meeting rooms. The servers offer a highly secure connecting with no third party data processing. The meeting room can only be access by the participating parties.

Before the video-consultation patient might have spoken about confidential matters, which they have not discussed with all healthcare providers, e.g. alcohol consumption or smoking, this could place the patient in a dilemma. Therefore, the intervention-guide for oncologist and GPs include a note about this.

## Discussion

Video-based consultation that brings the cancer patient, the GP and the oncologist together in the early phase of treatment may facilitate a sense of partnership that is powerful enough to improve the patient’s perception of intersectoral cooperation, continuity of cancer care and health-related quality of life. Beyond the RCT design, we intend to examine the consultations qualitative with a focus on the structure, content and benefits from this shared consultation. The method and description of this analysis are beyond the scope of this paper.

Based on our pilot study we believe that the study is feasible and intent to test the intervention in a pragmatic randomised controlled trial.

### Study limitations

A possible limitation of the study is the IIRS and CCCQ questionnaires. They were translated from English to Danish for this study. Although the translation was based on the guidelines for the process of cross-cultural adaptation of self-report measures proposed by Beaton et al. [39] they have not been validated in a Danish setting before study start. However, all other outcome measures have been validated and used before in a Danish mixed cancer-setting.

GPs can have patients in both groups leading to the possibility of a spillover effect. However, we anticipated this to be of minor significance since there are more than 500 GPs in the Region where recruitment is planned and the mean number of incident cancer patients per year per GP is eight. Therefore, most of the participating GPs would only have one patient enrolled in the study. Finally, the use of video-consultations is relatively new to most GPs in Denmark especially in regards to patient communication. Therefore, logistical concerns and technological barriers could be a limitation of the study.

## Additional files

Additional file 1: SPIRIT-PRO-Checklist. (DOCX 159 kb)

Additional file 2: English translation of the questionnaire containing the primary outcome “global assessment of inter-sectorial cooperation” in Word format. (DOCX 28 kb)

## Data Availability

Not applicable.

## Abbreviations

CCCQ: Cancer Care Coordination Questionnaire
DSM-IV: Diagnostic and Statistical Manual of Mental Disorders, Fifth Edition
EORCT – QLQ C 30: European Organization for Research and Treatment Quality of Life Questionnaire C-30
EORCT INFO 25: European Organization for Research and Treatment Information Questionnaire 25
GAD-7: The Generalised Anxiety Disorder seven scale
GP: General practitioner
IIRS: Illness Intrusiveness Rating Scale
OPEN: Odense Patient Data Explorative Network, Department of Clinical Research, University of Southern Denmark and Odense University Hospital
PHQ-9: The Patient Health Questionnaire nine scale
RCT: Randomised controlled trial
SPIRIT-PRO: Standard Protocol Items: Recommendations for Interventional Trials – Patient-Reported Outcomes
