# Supplementary material for: Improving continuity by bringing the cancer patient, general practitioner and oncologist together in a shared video-based consultation – protocol for a randomised controlled trial
Source: BMC Fam Pract. 2019 Jun 25;20:86. doi: 10.1186/s12875-019-0978-8 (PMC6593592; doi:10.1186/s12875-019-0978-8)
Supplement: Supplementary file 1 — SPIRIT-PRO-Checklist. (DOCX 159 kb) [file 12875_2019_978_MOESM1_ESM.docx]

# SPIRIT-PRO Extension Checklist - Guidelines for Inclusion of Patient-Reported Outcomes in Clinical Trial Protocols

**Table. SPIRIT 2013 and SPIRIT-PRO Extension Checklist: Recommended Items to Address in a Clinical Trial Protocol by Calvert et al.**

| **SPIRIT Section** | **SPIRIT Item No** | **SPIRIT Item Description** | **SPIRIT-PRO ITEM No** | **SPIRIT-PRO Elaboration Item Description** | **Addressed on page No** |
| --- | --- | --- | --- | --- | --- |
| **Administrative Information** | | | | | |
| Title | 1 | Descriptive title identifying the study design, population, interventions, and if applicable trial acronym |  |  | 1 |
| Trial registration | 2a | Trial identifier and registry name (if not yet registered, name of intended registry) |  |  | 17-18 |
|  | 2b | All items from the World Health Organization Trial Registration Data Set |  |  | - |
| Protocol version | 3 | Date and version identifier |  |  | 1 |
| Funding | 4 | Sources and types of financial, material, and other support |  |  | 21 |
| Roles and responsibilities | 5a | Names, affiliations, and roles of protocol contributors | SPIRIT-5a-PRO Elaboration | Specify the individual (s) responsible for the PRO content of the trial protocol | 21 |
|  | 5b | Name and contact information for the trial sponsor |  |  | 21 |
|  | 5c | Role of study sponsor and funders, if any, in study design; collection, management, analysis, and interpretation of data; writing of the report; and the decision to submit the report for publication, including whether they will have ultimate authority over any of these activities |  |  | 21 |
|  | 5d | Composition, roles, and responsibilities of the coordinating center, steering committee, end-point adjudication committee, data management team, and other individuals or groups overseeing the trial, if applicable (see item 21a for data monitoring committee) |  |  | 21 |
| **Introduction** | | | | | |
| Background and rationale | 6a | Description of research question and justification for undertaking the trial, including summary of relevant studies (published and unpublished) examining benefits and harms for each intervention | SPIRIT-6a-PRO extension | Describe the PRO-specific research question and rationale for PRO assessment and summarize PRO findings in relevant studies. | 4-6 |
|  | 6b | Explanation for choice of comparators |  |  | 7-8 |
| Objectives | 7 | Specific objectives or hypotheses | SPIRIT-7-PRO extension | State specific pro objectives of hypotheses (including relevant PRO concepts/domains) | 5-6 |
| Trial design | 8 | Description of trial design, including type of trial (eg, parallel group, crossover, factorial, single group), allocation ratio,and framework (eg, superiority, equivalence,noninferiort, exploratory) |  |  | 6 |
| **Methods: Participants, Interventions and Outcomes** | | | | | |
| Study setting | 9 | Description of study settings (eg, community clinic, academic hospital) and list of countries where data will be collected; reference to where list of study sites can be obtained |  |  | 6 |
| Eligibility criteria | 10 | Inclusion and exclusion criteria for participants; if applicable eligibility criteria for study centers and individuals who will perform the interventions (eg., surgeons, psychotherapist) | SPIRIT-10-PRO Extension | Specify any PRO-specific eligibility criteria (eg. Language/reading requirements of prerandomization completion of PRO). If PRO will not be collected from the entire study sample, provide a rationale and describe the method for obtaining the PRO subsample. | 6-7 |
| Interventions | 11a | Interventions for each group with sufficient detail to allow replication, including how and when they will be administered. |  |  | 7-8 |
|  | 11b | Criteria for discontinuing or modifying allocated interventions for a given trial participant (eg, drug dose change in response to harms, participant request, or improving/worsening disease) |  |  | 15 |
|  | 11c | Strategies to improve adherence to intervention protocols and any procedures for monitoring adherence (eg, drug tablet return, laboratory tests) |  |  | 12-13 |
|  | 11d | Relevant concomitant care and interventions that are permitted or prohibited during the trial |  |  | 7 |
| Outcomes | 12 | Primary, secondary, and other outcomes, including the specific measurement variable (eg, systolic blood pressure), analysis metric (eg, change from baseline, final value, time to event), method of aggregation (eg, median, proportion), and time point for each outcome; explanation of the clinical relevance  of chosen efficacy and harm outcomes is strongly recommended | SPIRIT- 12-PRO  Extension | Specify the PRO concepts/domains used to evaluate the intervention (eg, overall health-related quality of life, specific domain, specific symptom) and, for each one, the analysis metric  (eg, change from baseline, final value, time to event) and the principal time point or period of interest. | 8-11 |
| Participant | 13 | Time schedule of enrollment, interventions (including any run-ins and washouts), assessments, and visits for participants; a schematic diagram is highly recommended (se figure in CHAN et al) | SPIRIT-13-PRO Extension | Include a schedule of PRO assessments, providing a rationale for the time points, and justifying if the initial assessments is not prerandomization. Specify time windows, whether PRO collection is prior to clinical assessments, and, if using multiple questionnaires, whether order of administration will be standardized | 11 and  Figure 2+3 |
| Sample size | 14 | Estimated number of participants needed to achieve study objectives and how it was determined, including clinical and statistical assumptions supporting any sample size calculations | PIRIT-14-PRO Elaboration | When a PRO is the primary end point, state the required sample size (and how it was determined) and recruitment target (accounting for expected loss to follow-up). If sample size is not established based on the PRO end point, the discuss the power of the principal PRO analyses | 12 |
| Recruitment | 15 | Strategies for achieving adequate participant enrolment to reach target sample size |  |  | 12 |
| **Methods: Assignment of Interventions (for Clinical Trials)** | | | | | |
| Allocation | | | | | |
| Sequence generation | 16a | Method of generating the allocation sequence (eg, computer-generated random numbers), and list of any factors for stratification. To reduce predictability  of a random sequence, details of any planned restriction (eg, blocking) should be provided in a separate document that is unavailable to those who enroll participants or assign interventions. |  |  | 13-14 and Figure 3 |
| Allocation concealment mechanism | 16b | Mechanism of implementing the allocation sequence (eg, central telephone; sequentially numbered, opaque, sealed envelopes), describing any steps  to conceal the sequence until interventions are assigned |  |  | 13-14 and Figure 3 |
| Implementation | 16c | Who will generate the allocation sequence, who will enroll participants, and who will assign participants to interventions |  |  | 13-14 |
| Blinding | 17a | Who will be blinded after assignment  to interventions (eg, trial participants, care providers, outcome assessors, data analysts) and how. |  |  | 14 |
|  | 17b | If blinded, circumstances under which unblinding is permissible and procedure for revealing a participant’s allocated intervention during the trial. |  |  | - |
| **Methods: Data Collection, Management, and Analysis** | | | | | |
| Data collection methods | 18a | Plans for assessment and collection of outcome, baseline, and other trial data, including any related processes to promote data quality (eg, duplicate measurements, training of assessors) and description of study instruments (eg, questionnaires, laboratory tests) along with their reliability and validity, if known; reference to where  data collection forms can be found, if not in the protocol | SPIRIT-18a-PRO Extension | Justify the PRO instrument to be used and describe domains, number of items, recall period, and instrument scaling and scoring (eg, range and direction of scores indicating a good or poor outcome). Evidence of PRO instrument measurement properties, interpretation guidelines, and patient acceptability and burden should be provided or cited if available, ideally in the population of interest. State whether the measure will be used in accordance with any user manual and specify and justify deviations if planned. | 8-11, 16 and  Figure 2 |
|  |  |  | SPIRIT-18a(ii)-PRO Extension | Include a data collection plan outlining the permitted mode(s) of administration (eg, paper, telephone, electronic, other) and setting (eg, clinic, home, other). | 16 and Figure 2 |
|  |  |  | SPIRIT-18a(iii)-PRO Extension | Specify whether more than 1 language version will be used and state whether translated versions have been developed  using currently recommended methods. | 15 and 11 for the translation |
|  |  |  | SPIRIT-18a(iv)-PRO Extension | When the trial context requires someone other than a trial participant to answer on his or her behalf (a proxy-reported outcome), state and justify the useof a proxy respondent. Provide or cite evidence of the validity of proxy assessment if available. | - |
|  | 18b | Plans to promote participant retention and complete follow-up, including list of any outcome data to be collected for participants who discontinue or deviate from intervention protocols | SPIRIT-18b(i)-PRO Extension | Specify PRO data collection and management strategies for minimizing avoidable missing data. | 15 |
|  |  |  | SPIRIT-18b(ii)-PRO Extension | Describe the process of PRO assessment for participants who discontinue or deviate from the assigned  Intervention protocol. | 15 |
| Data management | 19 | Plans for data entry, coding, security, and storage, including any related processes to promote data quality (eg, double data entry; range checks for data values); reference to where details of data management procedures can be found, if not in the protocol |  |  | 16 |
| Statistical Methods | 20a | Statistical methods for analyzing primary and secondary outcomes. Reference to where other details of the statistical analysis plan can be found, if not in the protocol | SPIRIT-20a-PRO extension | State PRO analysis methods, including any plans for addressing multiplicity/type I (alpha) error. | 16 |
|  | 20b | Methods for any additional analyses (eg, subgroup and adjusted analyses) |  |  | 16 |
|  | 20c | Definition of analysis population relation to protocol nonadherence (eg. As randomized analysis) and any statistical methods to handle missing data (eg. Multiple imputation) | SPIRIT-20c-PRO Extension | State how missing data will be described and outline the methods for handling missing items or entire assessments (ge. Approach to imputation and sensitivity analyses) | 16 |
| **Methods: Monitoring** | | | | | |
|  | 21a | Composition of data monitoring committee; summary of its role and reporting structure; statement of whether it is independent from the sponsor and competing interests; and reference to where further details about its charter can be found, if not  in the protocol (alternatively, an explanation of why a data monitoring committee  is not needed) |  |  | 17 |
|  | 21b | Description of any interim analyses and stopping guidelines, including who will have access to these interim results and make the final decision to terminate the trial |  |  | 17 |
| Harms | 22 | Plans for collecting, assessing, reporting, and managing solicited and spontaneously reported adverse events and other unintended effects of trial interventions or trial conduct | SPIRIT-22-PRO Extension | State whether or not PRO data will be monitored during the study to inform the clinical care of individual trial participants and, if so, how this will be managed in a standardized way. Describe how this process will be explained to participants; eg, in the participant information sheet and consent form. | 17 |
| Auditing | 23 | Frequency and procedures for auditing trial conduct, if any, and whether the process will be independent from investigators and sponsor(s) |  |  | 17 |
| **Ethics and Dissemination** | | | | | |
| Research ethics approval | 24 | Plans for seeking research ethics committee/institutional review board approval |  |  | 17-18+20 |
| Protocol Amendments | 25 | Plans for communicating important protocol modifications (eg, changes to eligibility criteria, outcomes, analyses) to relevant parties (eg, investigators, research ethics committees/institutional review boards, trial participants, trial registries, journals, regulators) |  |  | 18 |
| Consent or assent | 26a | Who will obtain informed consent or assent from potential trial participants or authorized surrogates and how (see item 32) |  |  | 18 |
|  | 26b | Additional consent provisions for collection and use of participant data and biological specimens in ancillary studies, if applicable |  |  | - |
| Confidentiality | 27 | How personal information about potential and enrolled participants will be collected, shared, and maintained to protect confidentiality before, during, and after the trial |  |  | 18-19 |
| Declaration of interests | 28 | Financial and other competing interests for principal investigators for the overall trial and each study site |  |  | 20 |
| Access to data | 29 | Statement of who will have access to the final trial data set and disclosure of contractual agreements that limit such access for investigators |  |  | 18-19 |
| Ancillary and posttrial care | 30 | Provisions, if any, for ancillary and posttrial care and for compensation to those who are harmed by trial participation |  |  | 19 |
| Dissemination Policy | 31a | Plans for investigators and sponsor(s) to communicate trial results to participants, health care professionals, the public, and other relevant groups (eg, via publication, reporting in results databases, or other data-sharing arrangements), including any publication restrictions |  |  | 19 |
|  | 31b | Authorship eligibility guidelines and any intended use of professional writers |  |  | - |
|  | 31c | Plans, if any, for granting public access to the full protocol, participant-level data set, and statistical code |  |  | - |

The SPIRIT checklist is copyrighted by the SPIRIT Group under the Creative Commons “Attribution-NonCommercial-NoDerivs 3.0 Unported” license and is reproduced with permission.
