# Supplementary material for: Improving continuity by bringing the cancer patient, general practitioner and oncologist together in a shared video-based consultation – protocol for a randomised controlled trial
Source: BMC Fam Pract. 2019 Jun 25;20:86. doi: 10.1186/s12875-019-0978-8 (PMC6593592; doi:10.1186/s12875-019-0978-8)
Supplement: Supplementary file 2 — English translation of the questionnaire containing the primary outcome “global assessment of inter-sectorial cooperation” in Word format. (DOCX 28 kb) [file 12875_2019_978_MOESM2_ESM.docx]

**Patients’ attitude to the health care service**

**G The following questions are about your healthcare program**

This section deals with your assessment of your health care services. Therefore, it is essential that you, in your answer, try to assess only how you experience the help of the health service and do not think about the support and help you have received from your family and friends.

|  | (Tick each line) | | | | | | |
| --- | --- | --- | --- | --- | --- | --- | --- |
|  | *Look back on the last 3 months and mark how you agree with the following statements.* | Strongly agree | Agree | Neutral | Disagree | Strongly disagree | Not relevant |
| a | After I came out of hospital I sometimes felt as though I had been left “in limbo” | 1◼ | 2◼ | 3◼ | 4◼ | 5◼ | 6◼ |
| b | After I came out of hospital I knew who to go to for help and advice | 1◼ | 2◼ | 3◼ | 4◼ | 5◼ | 6◼ |
| c | I wish I had been given a little more help in coping with the medical side of my condition when I came out of hospital | 1◼ | 2◼ | 3◼ | 4◼ | 5◼ | 6◼ |
| d | I wish I had been given a little more help in coping with my worries when I came out of hospital | 1◼ | 2◼ | 3◼ | 4◼ | 5◼ | 6◼ |
| e | I would have liked to have been given more information about what to expect when I came out of hospital | 1◼ | 2◼ | 3◼ | 4◼ | 5◼ | 6◼ |
| f | The hospital staff could have done more to make sure that I would be able to cope when I went home | 1◼ | 2◼ | 3◼ | 4◼ | 5◼ | 6◼ |
| g | I have had enough information about my progress since I came out of hospital | 1◼ | 2◼ | 3◼ | 4◼ | 5◼ | 6◼ |
| h | Altogether I have found things concerning my care after leaving the hospital that could be improved | 1◼ | 2◼ | 3◼ | 4◼ | 5◼ | 6◼ |

**H The following questions are about how you experience the collaboration between hospital doctors at the Department of Oncology, Vejle Hospital and your general practitioner.**

|  | (Tick each line) | | | | | | |
| --- | --- | --- | --- | --- | --- | --- | --- |
|  | *Look back on the last 3 months and mark how you agree with the following statements.* | Strongly agree | Agree | Neutral | Disagree | Strongly disagree | Not relevant |
| a | I wished there would be better collaboration between the hospital doctors and my general practitioner | 1◼ | 2◼ | 3◼ | 4◼ | 5◼ | 6◼ |
| b | I felt confident about the cooperation between my general practitioner and the hospital doctors | 1◼ | 2◼ | 3◼ | 4◼ | 5◼ | 6◼ |
| c | I got the impression that the hospital doctors left out my general practitioner | 1◼ | 2◼ | 3◼ | 4◼ | 5◼ | 6◼ |
| d | The hospital doctors have kept my general practitioner informed of my progress | 1◼ | 2◼ | 3◼ | 4◼ | 5◼ | 6◼ |
| e | *Altogether I have been satisfied with the cooperation between my general practitioner and the hospital doctors | 1◼ | 2◼ | 3◼ | 4◼ | 5◼ | 6◼ |

* The primary study outcome is the single item “global assessment of inter-sectorial cooperation”.

**I The following questions are about your contact with your general practitioner**

You have to think back on the contacts you have had with your general practitioner for the last 3 months. By contacts is meant any connection to your family doctor (phone consultation, mail correspondence, visits to the doctor, sick visits or any other conversation).

|  | (Tick each line) | | | | | | |
| --- | --- | --- | --- | --- | --- | --- | --- |
|  | *Look back on the last 3 months and mark how you agree with the following statements.* | Strongly agree | Agree | Neutral | Disagree | Strongly disagree | Not relevant |
| a | I felt the general practitioner told me everything that I wanted to know | 1◼ | 2◼ | 3◼ | 4◼ | 5◼ | 6◼ |
| b | Sometimes I thought that the general practitioner did not see my condition as being very important | 1◼ | 2◼ | 3◼ | 4◼ | 5◼ | 6◼ |
| c | I came away from the general practitioner visits with some of my questions unanswered | 1◼ | 2◼ | 3◼ | 4◼ | 5◼ | 6◼ |
| d | I did not get enough advice on my conditions | 1◼ | 2◼ | 3◼ | 4◼ | 5◼ | 6◼ |
| e | I felt the general practitioner told me everything that I wanted to know | 1◼ | 2◼ | 3◼ | 4◼ | 5◼ | 6◼ |
| f | My general practitioner has supported me during my illness | 1◼ | 2◼ | 3◼ | 4◼ | 5◼ | 6◼ |
| g | I felt confident about my general practitioner knowledge of my disease | 1◼ | 2◼ | 3◼ | 4◼ | 5◼ | 6◼ |
| h | My general practitioner is very good at listening to me | 1◼ | 2◼ | 3◼ | 4◼ | 5◼ | 6◼ |
| i | My general practitioner takes the time to listen to me | 1◼ | 2◼ | 3◼ | 4◼ | 5◼ | 6◼ |
| j | My general practitioner has enough knowledge of my disease | 1◼ | 2◼ | 3◼ | 4◼ | 5◼ | 6◼ |
| k | My general practitioner has enough knowledge of the treatment of my disease | 1◼ | 2◼ | 3◼ | 4◼ | 5◼ | 6◼ |
| l | My general practitioner has enough knowledge of the side effects of my treatment | 1◼ | 2◼ | 3◼ | 4◼ | 5◼ | 6◼ |
| m | Altogether my general practitioner could have supported me better during my illness | 1◼ | 2◼ | 3◼ | 4◼ | 5◼ | 6◼ |
